# NeuroBioSense: A multidimensional dataset for neuromarketing analysis

**DOI:** 10.1016/j.dib.2024.110235

**Published:** 2024-02-27

**Authors:** Büşra Kocaçınar, Pelin İnan, Ela Nur Zamur, Buket Çalşimşek, Fatma Patlar Akbulut, Cagatay Catal

**Affiliations:** a Department of Computer Engineering, Istanbul Kültür University, Istanbul, Turkey; b Department of Software Engineering, Istanbul Kültür University, Istanbul, Turkey; c Department of Computer Science and Engineering, Qatar University, Doha, Qatar

**Keywords:** Neuromarketing, Biosignals, Image processing

## Abstract

In the context of neuromarketing, sales, and branding, the investigation of consumer decision-making processes presents complex and intriguing challenges. Consideration of the effects of multicultural influences and societal conditions from a global perspective enriches this multifaceted field. The application of neuroscience tools and techniques to international marketing and consumer behavior is an emerging interdisciplinary field that seeks to understand the cognitive processes, reactions, and selection mechanisms of consumers within the context of branding and sales. The NeuroBioSense dataset was prepared to analyze and classify consumer responses. This dataset includes physiological signals, facial images of the participants while watching the advertisements, and demographic information. The primary objective of the data collection process is to record and analyze the responses of human subjects to these signals during a carefully designed experiment consisting of three distinct phases, each of which features a different form of branding advertisement. Physiological signals were collected with the Empatica e4 wearable sensor device, considering non-invasive body photoplethysmography (PPG), electrodermal activity (EDA), and body temperature sensors. A total of 58 participants, aged between 18 and 70, were divided into three different groups, and data were collected. Advertisements prepared in the categories of cosmetics for 18 participants, food for 20 participants, and cars for 20 participants were watched. On the emotion evaluation scale, 7 different emotion classes are given: Joy, Surprise, anger, disgust, sadness, fear, and neutral. This dataset will help researchers analyse responses, understand and develop emotion classification studies, the relationship between consumers and advertising, and neuromarketing methods.

Specifications Table

**Table:** 

Subject	Computer Science, Data Science, Psychology
Specific subject area	Emotion detection, Signal classification, Signal Processing, Video classification, Video Processing, Image classification, Image Processing
Data format	Raw Signal Pre-processed Signal Labelled Videos
Type of data	Signals Videos Table
Data collection	Several weeks were devoted to the extensive acquisition of physiological and visual data from a variety of participants. The duration of data collection for each subject was determined based on the durations of the presented advertisements and the time required for participants to indicate their emotional states.18 participants viewed a total of 15 advertisements in the “cosmetics and fashion” category, while 20 participants viewed 10 advertisements in the “cars and technology” category. In addition, 10 advertisements representing the category “food and market” were observed by a distinct group of 20 people. In total, the cumulative duration of all video stimuli was between 10 and 15 min. However, the duration of the session as a whole was lengthened as inquiries were administered after each advertisement to collect participantsʼ opinions. During the data collection sessions, great care was taken to ensure that the participants were entirely engrossed in the advertisements in a serene and comfortable setting. Subsequently, emotion labels were ascribed to the biosignal data based on the analysis of the recorded videos, images, and the information gathered from the demographic questions that participants answered during the advertisement viewing sessions. These questions are related to emotional assessments of the advertisements and the submission of germane demographic data. Within the video dataset, facial expressions were meticulously recorded, with an emphasis on detecting the presence of the seven primary emotions, namely anger, disgust, fear, joy, sadness, surprise, and neutral, based on the expressive indicators of the participants in the provided images.
Data source location	Institution: Istanbul Kultur University, Department of Computer Engineering • City/Town/Region: İstanbul, Bakırköy • Country: Turkiye • Latitude and longitude (and GPS coordinates, if possible) for collected samples/data: 41° 5′ 7.3284′' N 29° 2′ 22.3836′' E
Data accessibility	Repository name: Mendeley Data Data identification number: 10.17632/7md7yty9sk.1 Direct URL to data: https://data.mendeley.com/datasets/7md7yty9sk/2

## 1 Value of the Data

• The NeuroBioSense dataset is extremely valuable to the scientific community because it combines physiological signals and facial expressions to provide a comprehensive comprehension of emotional responses to branding advertisements, which is essential for neuromarketing research.
• The dataset contains a variety of advertisements from various categories, ministering to the global and multicultural nature of consumer behavior, and thus providing a rich and multidimensional perspective for the study of consumer decision-making processes.
• With data collected from three groups of volunteers spanning an age range of 18–70, this dataset provides the opportunity to investigate how emotional responses to marketing stimuli may vary across demographic groups.
• The inclusion of a demographic information alongside emotional assessments enhances the analytic potential of the dataset, allowing researchers to investigate the influence of individual characteristics on emotional responses, thereby facilitating the development of personalized marketing strategies.
• Using the Empatica e4 wearable sensor device to record non-invasive physiological signals such as photoplethysmography (PPG), electrodermal activity (EDA), and body temperature, is highly pertinent to the advancement of neuromarketing methodologies.
• Understanding the neural basis of consumer-advertising relationships through this dataset can provide businesses, marketers, and advertisers with novel insights for developing effective neuromarketing strategies that resonate with the affective states of consumers.

## 2 Data Description

**Fig. 1:**
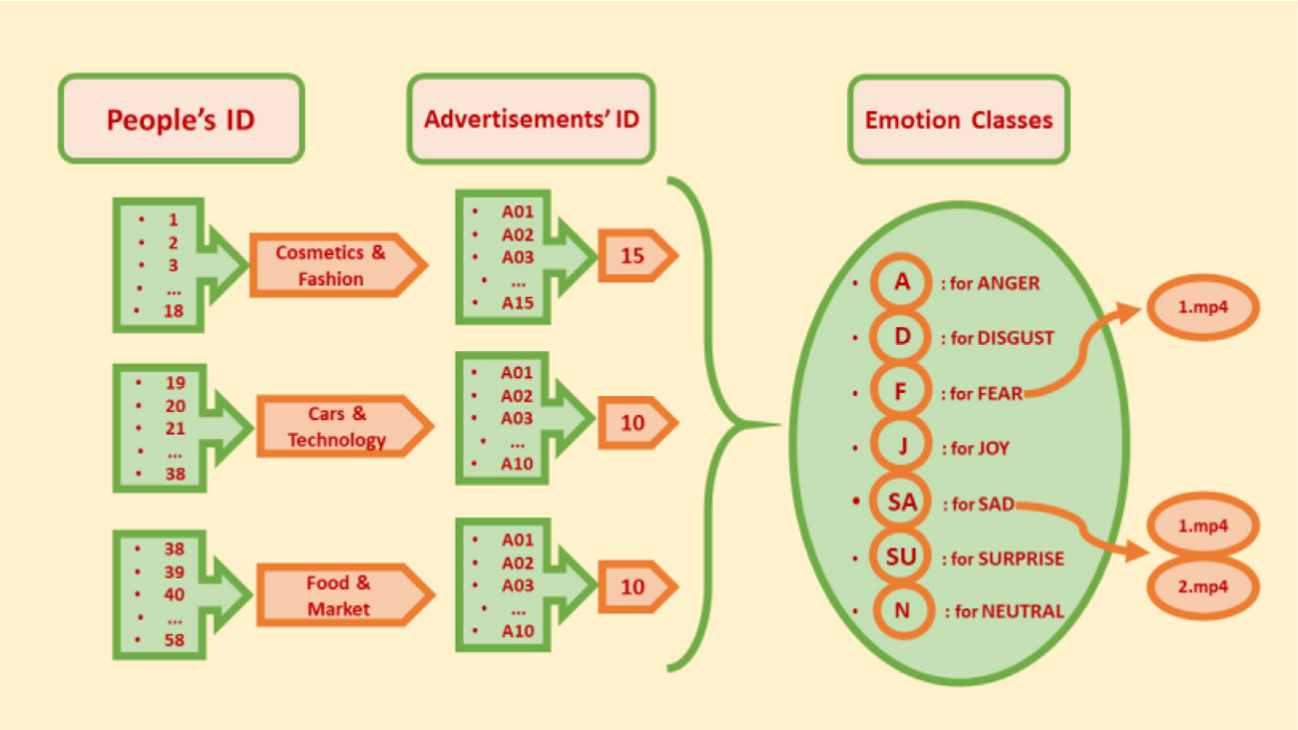
The structure of advertisement categories subfolder.
c. Biosignal Subfolder
  i. Pre-Processed Files: It contains the csv file containing BVP, EDA, TEMP, ACC signals and emotion tags at different frequencies: 32-Hertz.csv, 4-Hertz.csv, 64-Hertz.csv.
  ii. Raw Files: It 4 different signal folders: ACC, BVP, EDA, TEMP. Inside these folders are raw signal information collected from the participants, depending on the frequency value of each signal.

The dataset is organized in a structured way, with multiple folders, subfolders, and files containing both raw and processed data. The subsequent sections provide an overview of the structure of the dataset:

a. Participant Data Subfolder: Contains individual participant data files, each designated with a unique participant identifier.
  i. Participant_demographic_information.csv: Demographic information of each participant, including age, gender, and other relevant details and participant responses to the emotion appraisal scale.
b. Advertisement Categories Subfolder: This subfolder contains subfolders organized by the category of advertisements viewed. The structure visualized in Fig. 1.
  i. Cosmetics_and_Fashion_Subfolder: Contains recordings and relevant data files for the advertisements for cosmetics and fashion.
    1. It contains 18 folders according to the ID of each participant watching the advertisement in this category.
      a. Each folder also contains 15 folders representing each ad they watch. For example, A01, A02... These folders contain tagged video recordings.
  ii. Car_and_Technology_Subfolder: Individual car and technology advertisement related files.
    1. It contains 20 folders according to the ID of each participant watching the advertisement in this category.
      a. Each folder also contains 10 folders representing each ad they watch. For example, A01, A02... These folders contain tagged video recordings.
  iii. Food_and_Market_Subfolder: Individual food and market advertisement related files.
    1. It contains 20 folders according to the ID of each participant watching the advertisement in this category.
      a. Each folder also contains 10 folders representing each ad they watch. For example, A01, A02... These folders contain tagged video recordings.

“Participant_demographic_information.csv” file contains the questions answers of 58 participants. The data description for the demographic information dataset is shown in Table 1.

**Table 1:** Demographic information dataset description.

Data	Data type	Data description
Record id	Integer	All the id of the rar files that hold all the signal information of the participants
Subject id	Integer	Participant's id
Gender	String	Participant's gender
Age	Integer	Participant's age
Ad Code	Integer	Advertising numbers
Duration	Integer	The hour and minute the ad started to watch
Emotions (%)	String\Integer	Evaluation of 6 main emotions as a percentage
Interested in	Boolean	Whether there is interest in the advertisement

**Fig. 2:**
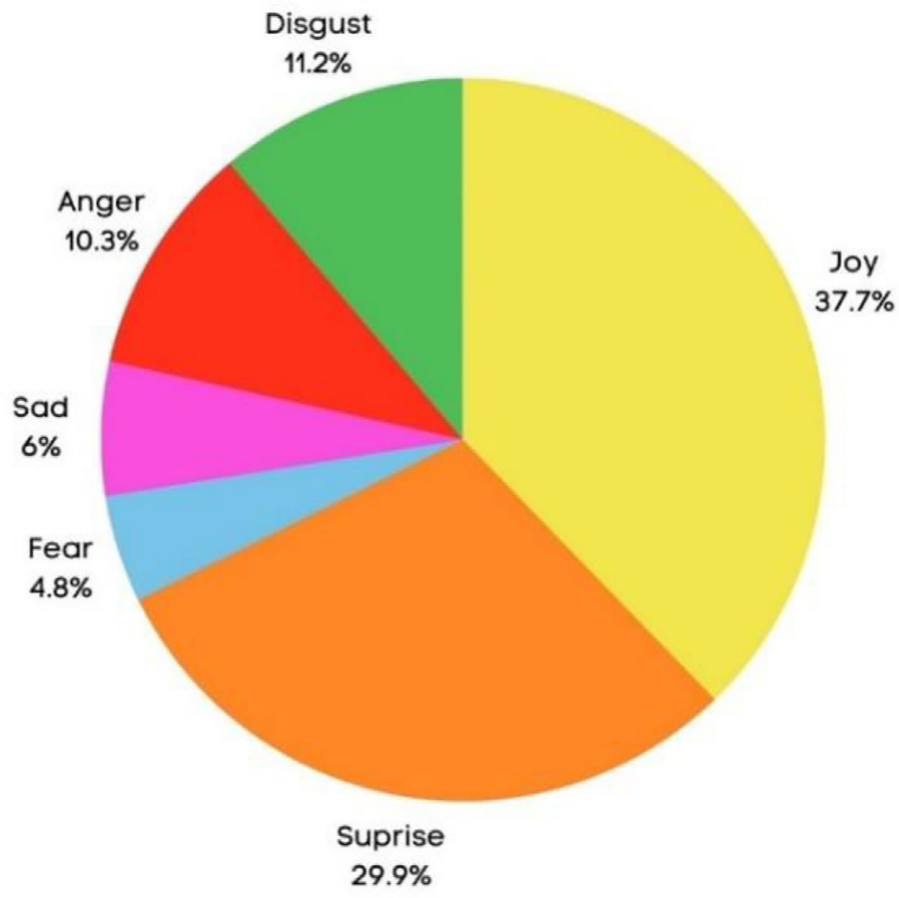
Emotion rates across the entire participants.

In a broad sense, the provided graphical representation, depicted in Fig. 2, illustrates the comprehensive dispersion of emotions across the entire subject cohort. It is evident that among the range of emotions experienced, joy emerged as the predominant sentiment, accounting for approximately 37.7% of the overall emotional responses.

## 3 Experimental Design, Materials and Methods

Neuromarketing uses various analyzes to emotionally understand consumer behavior beyond traditional marketing and advertising research. Various physiological signals, biometric data and psychological data are used for analysis. According to the results, it can be understood how and from what the consumer is emotionally affected in the purchase decision [1,2]. Investigating the effects of emotion detection and purchasing with facial expressions is part of this field and various studies are being conducted. Moreover, with these research results, advertising campaigns can be planned more accurately [3].

### 3.1 Study procedure

**Fig. 3:**
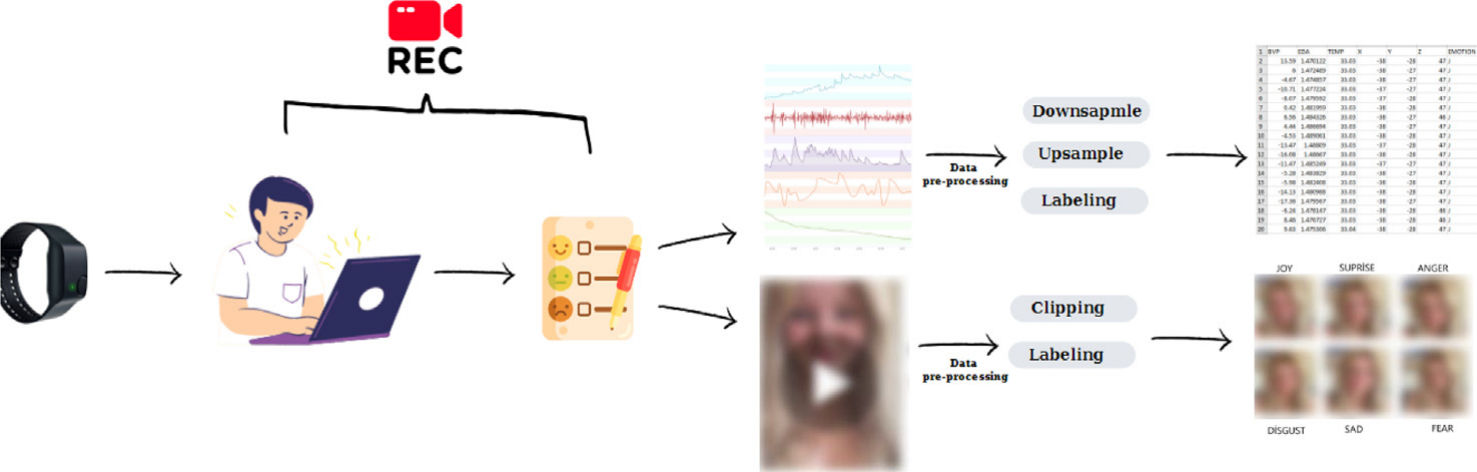
Procedures for acquiring and processing data.

The data collection process consists of three steps, as shown in Fig. 3. Firstly, the participants were equipped with Empatica E4 wristbands which is used in many studies in the similar field [4], [5], [6]. 35 distinct advertisements, including 15 in cosmetics, 10 in cars, and 10 in food categories, were selected and prepared for the data collection phase. On average, each person watched 10 social media ads. At the end of each advertisement, participants were asked about the emotions evoked by the commercial and whether it captured their interest. Simultaneously, participantsʼ facial expressions were recorded using a mobile phone. Subsequently, data containing participantsʼ facial expressions were collected from these recordings. Additionally, using the wristbands, biological signal data of the participants were collected. Approximately, 15 min of biological data were obtained from each individual.

### 3.2 Study population

**Fig. 4:**
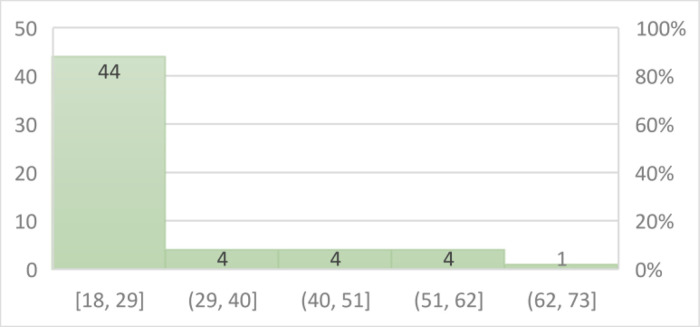
Age Distribution of participants.

To capture a diverse range of perspectives, a total of 58 participants spanning a wide age range, from 18 to 70 years old, were purposefully chosen. Fig. 4 shows the age distribution of our participants.

**Fig. 5:**
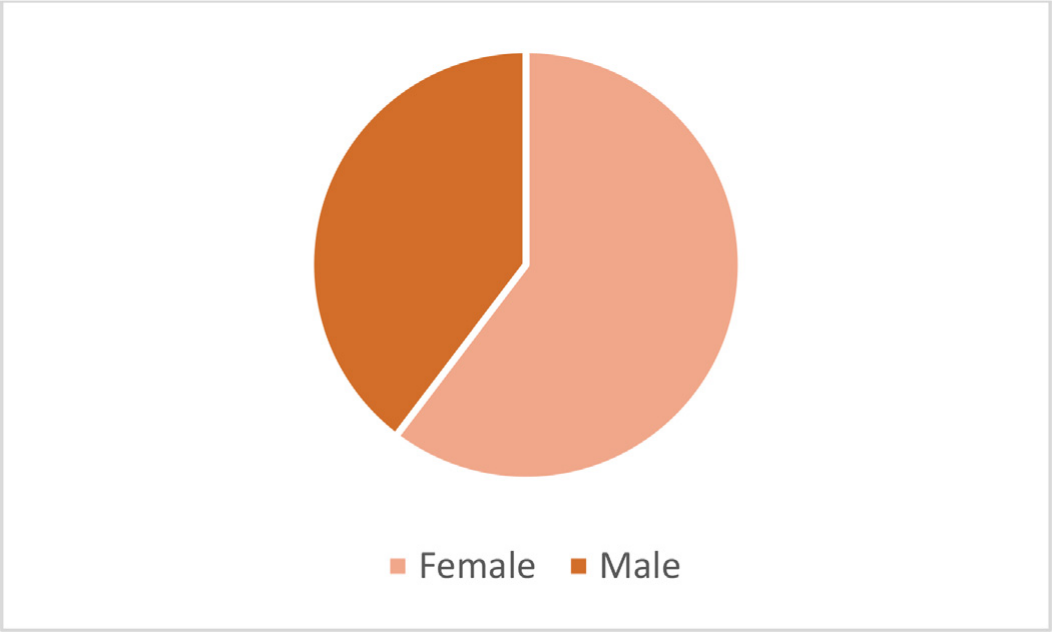
Gender distribution of dataset.

Among these participants, 18 participants were assigned to watch cosmetic ads, 20 participants focused on food ads, and another 20 participants focused on car ads. As shown in Fig. 5, our participants, to whom the advertisements are watched, consist of 60% female and 40% male. However, due to the uneven distribution, an assessment cannot be made based on age.

### 3.3 The biosignal data

In the data collection phase, only four of the data collected using the E4 wristband were utilized: blood volume pulse (BVP), electrodermal activity (EDA), body temperature (TEMP) and 3-axis accelerometer data (ACC).

Biological data was obtained from a total of 58 participants wearing the E4 wristband throughout the entire duration of the advertising sessions. Each participant provided signal data for an average of 20 minutes. These data are generally available because the wristband was stopped once all the advertisements were viewed by the participants.

The BVP, EDA, and TEMP data are contained in a single column consisting of thousands of rows, while the ACC signal data consists of three columns and also thousands of rows. The EDA and TEMP data are sampled at a rate of 4 samples per second, while the BVP data is sampled at 64 samples per second, and the ACC data is sampled at 32 samples per second. Additionally, due to significant individual variability in biological signal data, it is not possible to provide an average number of rows. Therefore, it was necessary to segment the acquired thousands of rows of data to align them with the durations of the advertisements watched by the participants. Following this segmentation process, we obtained the collected biological signals for each advertisement and labelled them based on the participants' responses.

After determining the ad times, the start time of each previously recorded ad was used to determine which ad each participant watched per second. Using this information, the entire dataset was divided and organized on an advertisement basis. Separate folders for each signal (BVP, EDA, TEMP, and ACC) were created. For ACC, like the other signals, we divided the entire NeuroBioSense dataset based on each advertisement's start times and durations for each participant. However, the split CSV files were stored under three folders (X, Y, and Z) for ACC. Consequently, the folder path took the form of ACC/A02/X/1.csv or ACC/A02/Y/1.csv.

### 3.4 The facial image data

The videos of the facial expressions of the participants while watching the advertisement were recorded using mobile phones. The collected data was then manually segmented. During the segmentation process, we considered the participantʼs facial expression containing emotions. The segmented videos have an average duration of around 4 s. Since an advertisement can contain multiple emotions, we divided a video into several parts and performed folder categorization. This folder categorization allowed us to complete the labelling process. As a result, we obtained 1288 labelled videos from a total of 670 videos.

Each advertisement video was analysed to identify the sections featuring the 7 basic emotions of individuals (Joy(J), Sadness (SA), Anger(A), Disgust(D), Surprise (SU), Neutral(N), and Fear(F)), and these sections were cut using Adobe Premiere Pro^1^ video editing application to be no longer than 6 seconds. Most videos were split into multiple parts because each video could contain more than one emotion. Videos were initially saved in the MOV format with a resolution of 828 × 1792 pixels before processing. After the cutting process was completed, the videos were saved in MP4 format with a resolution of 720 × 1280 pixels. The videos were saved in folders corresponding to the emotion shown in the advertisement video of the relevant individual. If two reactions were detected (Joy and Sadness) in the video taken while watching the 6th advertisement of person 4, these emotions were cut from the video and saved under folders named ``J'' and ``SA'' respectively. Thus, the folder structure became ``4/A06/J/1.MP4'' and ``4/A06/SA/1.MP4.''

### 3.5 Data resampling

To perform data pre-processing on NeuroEmoResponse dataset, initially, the dataset is split into two sets: X (independent variables) and Y (dependent variable). X comprises signal columns, while Y corresponds to the ‘EMOTION’ column.

A data encoding process was applied to merge signals with different frequencies. The varying data rates of each signal were considered. For instance, while the BVP signal collected 64 data points per second, the EDA signal only had 4 data points per second. Therefore, the data needed to be equalized to ensure that all signals end at the same row count in the same Excel file.

To equalize the data points of the signals, linear interpolation was used in some steps, while averaging was applied in others. For example, to align the ACC signal with a frequency of 32 with the BVP signal, the average of the two available data points in BVP was calculated and written to a single row using code. This downsampling process reduced the data points of the BVP signal, which had a frequency of 64, to match the ACC signal's frequency of 32.

A different approach was applied for EDA and TEMP signals. Since these signals only collected 4 data points per second, upsampling was performed to match a frequency of 32. Linear interpolation was used for upsampling, generating synthetic data points between the existing data points and increasing the signal data by a factor of 8 to achieve 32 data points.

Similar upsampling and downsampling procedures were carried out for signals with other frequencies. As a result, all signal data with frequencies of 4, 32, and 64 were consolidated in three separate Excel files. This data encoding process resolved the data inconsistency arising from different signal frequencies, facilitating the analysis and evaluation process. The resulting dataset was created by merging signals with different frequencies and associating them with labelled emotional data.

In signal dataset pre-processing class counts of Y (EMOTIONS) are calculated, and the SMOTE technique is utilized to address dataset imbalance, resulting in X NEW and Y NEW. In emotion distribution of 4 Hertz dataset is given depending on the SMOTE.

**Fig. 6:**
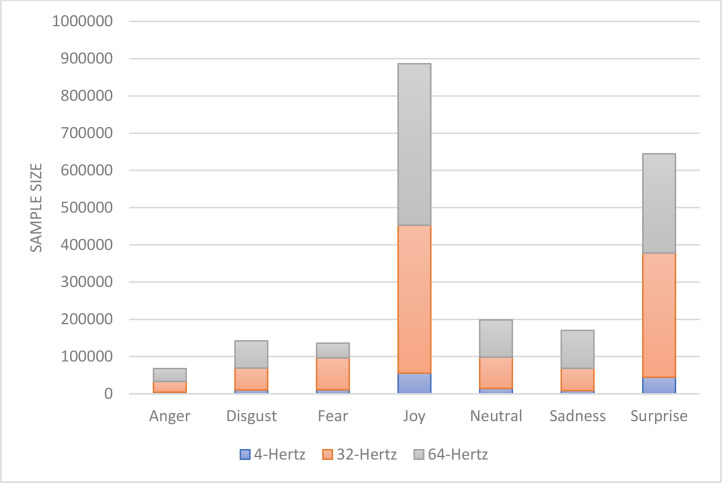
Distribution of emotion classes according to sampling frequencies.

Fig. 6 shows the emotion distributions in the dataset according to the frequencies after the upsampling and downsampling methods applied.

## Limitations

The study's shortcomings involve various crucial aspects: Initially, the limitations of the study were the limited sample size and the uneven distribution of participants across different age groups, especially with fewer individuals in the older age categories. These factors restricted the ability to thoroughly evaluate the effects of age on emotional reactions to the advertising. Furthermore, the limited generalizability of the findings was a result of the specific demographics and cultural backgrounds of the participants. Therefore, it is necessary to include a more diverse participant pool. Furthermore, the limited temporal resolution of the gathered physiological data may have hindered the ability to accurately capture quick changes in emotional states, which could have affected the accuracy of the correlations between physiological signals and transitory emotions. Furthermore, the interpretation of emotional correlations may have been complicated by external effects, such as ambient conditions or situational circumstances during data collection. Additionally, the study's exclusive emphasis on the influence of advertisements may restrict the understanding of emotional reactions in wider situations beyond the selected stimuli. Finally, the study's extensive investigation of emotions and their persistence across time may have been constrained by limits in the depth of emotion identification and the lack of longitudinal data. These limitations emphasize the importance of careful analysis and propose potential areas for further investigation to improve the comprehensiveness and range of evaluations of emotional responses in neuromarketing studies.

## Ethics Statement

Within the scope of the study, data were collected with the permission of the ethics committee of Istanbul Kultur University, with the decision dated 23.05.2022 and numbered 2022.107.

## CRediT authorship contribution statement

**Büşra Kocaçınar:** Data curation, Visualization, Investigation, Writing – original draft, Writing – review & editing. **Pelin İnan:** Data curation, Visualization, Investigation. **Ela Nur Zamur:** Data curation, Visualization, Investigation. **Buket Çalşimşek:** Data curation, Visualization, Investigation. **Fatma Patlar Akbulut:** Supervision, Conceptualization, Methodology, Investigation, Writing – review & editing. **Cagatay Catal:** Investigation, Writing – review & editing.

## Data Availability

NeuroBioSense (Original data) (Mendeley Data).

## Footnotes

1 Available at https://www.adobe.com/products/premiere.html.
